# Proof-Reader Gone Wrong

**Published:** 1895-02

**Authors:** 


### PROOF-READER GONE WRONG.

Our good friend the editor of the *Dominion Dental Journal* must have been absent from the sanctum when the December number was in preparation, or he would not have permitted the reader to give credit of authorship of "The New Era in Dental Practice," published in the September number of this journal, to "Professor Flagg, of Philadelphia." We have too much of an ever-present fear before us of errors to complain seriously, and would not disturb our serenity over this; but we feel regret that our contemporary should cast on the shoulders of the great apostle of the "New Departure" the burden of the mantle of the prophet of the "New Era," with all its possibilities.

To those inclined to follow the example of the *New Dominion Journal* and copy the article, they will confer a favor by giving credit to Dr. W. G. A. Bonwill, of Philadelphia.

